# Unmasking the Silent Struggle: The Impact of Psychological Distress and Social Support on Health-Related Quality of Life in Crohn’s and Ulcerative Colitis

**DOI:** 10.31662/jmaj.2025-0524

**Published:** 2026-04-24

**Authors:** Purvi Sukhwal, Ghazanfar Aftab, Minal Tariq, Daniah Hisham Al-Sammarraie, Alhasan Hisham Al-Sammarraie, Zainab Hisham Al-Sammarraie, Afnan Ahmed, Muhammad Saad Ullah, Abdulrahman AlQaderi, Maheen Haque, Hayian Omran

**Affiliations:** 1 Department of Cardiology, C.K. Birla Hospital, Jaipur, India; 2 Department of Medicine, Jinnah Hospital, Lahore, Pakistan; 3 Department of General Medicine, Maryam Nawaz Health Clinic (Basic Health Unit), Faisalabad, Pakistan; 4 Department of General Medicine, Tbilisi State Medical University, Tbilisi, Georgia; 5 Department of Medicine, Allama Iqbal Medical College, Lahore, Pakistan; 6 Department of Health & Population, Town Hospital, Multan, Pakistan; 7 Department of Internal Medicine, RAK Medical and Health Sciences University, Ras Al Khaimah, United Arab Emirates; 8 Department of Medicine, Dow International Medical College, Karachi, Pakistan; 9 Department of Internal Medicine, RAK Medical and Health Sciences University, Ras Al Khaimah, United Arab Emirates

**Keywords:** inflammatory bowel disease, Crohn’s disease, ulcerative colitis, psychological distress, social support, health-related quality of life

## Abstract

**Introduction:**

Inflammatory bowel disease (IBD) is a long-standing, recurrent disorder that affects not only physical health but also emotional well-being and social functioning, ultimately diminishing patients’ health-related quality of life (HRQL). This paper assessed the association between psychological distress and perceived social support and HRQL in people with Crohn’s disease (CD) and ulcerative colitis (UC).

**Methods:**

A cross-sectional survey was conducted among 504 adults with IBD in outpatient gastroenterology clinics. Participants completed measures of psychological distress, perceived social support, and HRQL. Correlation, independent-samples t-tests, one-way analysis of variance, and multiple linear regression were used to analyze the data.

**Results:**

There were significant negative correlations between psychological distress and social support (r = −0.53, p < 0.001) and between psychological distress and HRQL (r = −0.48, p < 0.001); however, there was a positive correlation between social support and HRQL (r = 0.62, p < 0.001). There was increased distress and poorer HRQL in females and younger adults. Patients with CD showed a higher level of distress and reduced HRQL compared to patients with UC. Regression revealed that HRQL was negatively predicted by psychological distress and disease activity, but positively predicted by social support and age.

**Conclusions:**

HRQL is negatively associated with greater distress and active disease, whereas greater social support is positively associated with well-being of patients with IBD. Psychosocial care is potentially effective in improving clinical management.

## Introduction

Inflammatory bowel disease (IBD), encompassing Crohn’s disease (CD) and ulcerative colitis (UC), refers to a long-term, relapsing inflammatory condition of the gastrointestinal tract ^(1)^. The incidence of IBD has plateaued in Western countries, including the USA and Germany, where values are above 0.3%, while this condition is steeply rising in Asia and other newly industrialized areas such as Brazil and Taiwan, both having estimated annual percent changes of up to +11% for CD and +15% for UC ^(2)^.

Social isolation and loneliness are major predictors of psychological well-being among patients with IBD, and disease activity mediates the relationship ^(3)^. There are high anxiety and depression with poor health-related quality of life (HRQL), often associated with body image and social function in patients with CD. Illness perceptions and stress also mediate the association between disease severity and psychological distress, highlighting the importance of integrated psychological care ^(4), (5)^.

Social support has been identified as a key protective factor that mitigates stress and enhances overall psychological well-being in patients with IBD, with greater economic security and higher levels of social support associated with lower distress and better HRQL in both UC and CD ^(6), (7)^. However, social support can have negative connotations when perceived as intrusive or unwelcome, resulting in social withdrawal, increased anxiety, and unwillingness to share experiences of illness ^(8)^.

HRQL is markedly impaired in patients with active IBD and may be worse in patients with CD than in those with UC ^(9)^. Psychological symptoms mediate the association among disease activity, social support, and quality of life (QOL) in patients with IBD; emotional well-being plays a critical role in managing and controlling this condition ^(10)^.

To our knowledge, few studies have assessed psychological distress, social support, and HRQL together within the same analytical framework. Furthermore, limited research has directly compared how these psychosocial variables interact in large outpatient samples across CD and UC ^(6), (7), (10)^.

### Rationale and objectives

CD and UC are chronic conditions that can have a significant impact on daily life. The chronicity of these diseases often impacts psychosocial health, including feeling stressed, sad, and anxious, which may affect global QOL. On the other hand, strong social support from family, friends, and peers can help patients cope and even feel more positive about their condition. Taking into account the mediating influence of psychological distress and the moderating influence of social support on adherence behaviors to illness, this research aims to investigate how these two factors combined are related to HRQL in patients with CD and UC.

The following are the objectives:

1. To investigate the level of psychological distress in patients with CD and UC.

2. To assess perceived social support in patients with IBD.

3. To investigate the relationship among psychological distress, social support, and HRQL in these patients.

4. To describe psychological and social variables, and to compare QOL between patients with CD and UC.

## Materials and Methods

### Study framework

In this study, we employed a cross-sectional questionnaire to investigate the effect of psychological distress and social support on HRQL in patients with CD and UC.

### Study setting and duration

The research was conducted at gastroenterology outpatient clinics in different cities. Data collection lasted five months, from February 2025 to July 2025.

### Study population

The study included individuals aged 18 years and older with a confirmed diagnosis of CD or UC. Participants who could read and comprehend the language in which the questionnaire was presented and who consented to participate were included. At the same time, patients with other chronic diseases, psychiatric diseases, hospitalizations, or acute disease flares at the time of data collection were excluded.

### Sampling technique and sample size

A convenience sampling strategy was used because patients were recruited consecutively during routine outpatient visits, and the number of eligible patients with IBD attending clinics was limited. Due to the use of non-probability sampling, a formal statistical sample size calculation was not applicable. All eligible patients present during the data collection period were invited to participate, resulting in a final sample of 504 participants.

### Data collection tools

#### Demographic form

Current disease activity was assessed via a single self-reported item: “How active is your IBD currently?” Response options were categorical: remission (no symptoms), mild symptoms, moderate symptoms, and severe symptoms, with higher categories indicating greater symptom severity. This single-item measure provided an estimate of disease activity at the time of data collection; however, it is not a validated clinical index, which is acknowledged as a limitation of the study. This form was newly designed for this study to obtain the relevant sociodemographic and clinical details of the participants.

#### Hospital Anxiety and Depression Scale

The Hospital Anxiety and Depression Scale (HADS) was employed to evaluate psychological distress. The HADS was established by Zigmond and Snaith in 1983 as a screening measure for anxiety and depression among patients in the hospital setting. It includes 14 items designed to measure anxiety (7) and depression (7). Responses are scored from 0 to 3 on a Likert scale, where higher scores are associated with higher levels of anxiety or depression. The total score ranges from 0 to 21 for each scale, with a score of 8 or greater on either subscale considered as a cut-point for potential anxiety or depression ^(11)^.

#### Multidimensional Scale of Perceived Social Support

Perceived social support among the participants was measured using the Multidimensional Scale of Perceived Social Support (MSPSS). MSPSS was created by Zimet et al. ^(12)^ in 1988 and includes 12 items which measure support from three sources: family, friends, and significant others. All items are rated on a 7-point scale (from 1 = strongly disagree to 7 = strongly agree), with higher total scores reflecting greater perceived social support (12-84) ^(12)^.

#### Short Inflammatory Bowel Disease Questionnaire

HRQL was assessed using the Short Inflammatory Bowel Disease Questionnaire (SIBDQ). Developed by Jowett et al. ^(13)^ in 2001, the SIBDQ is a 10-item measure of four domains (bowel symptoms, systemic symptoms, emotional function, and social function). Scores for each item range from 1 to 7 on a Likert scale (from 1 = serious problem to 7 = no problem at all), with higher scores indicating better HRQL. Scores can range from 10 to 70, with higher scores indicating a better HRQL ^(13)^.

### Data collection procedure

Once ethical approval was received, patients were recruited from their usual clinic setting. They were informed about the study objective and written consent was obtained from them. Questionnaires were self-administered. Trained supervisors assisted only for reading or clarifying items, when necessary, but were instructed not to influence responses in any way to minimize response bias.

### Data analysis

All analyses were performed using IBM SPSS Statistics version 26 (IBM Corp., Armonk, NY, USA). Descriptive statistics (means, standard deviations, frequencies, and percentages) were used to summarize demographic and clinical variables, and the normality of continuous data was assessed using the Kolmogorov-Smirnov and Shapiro-Wilk tests. Pearson’s correlation examined associations among psychological distress, perceived social support, and HRQL, while group differences were evaluated using independent-samples t-tests and one-way analysis of variance. Univariate and multivariable linear regression analyses were conducted to identify factors associated with HRQL. Psychological distress, perceived social support, age, sex, disease type, disease duration, and disease activity were included in the multivariable model based on theoretical relevance. All variables were entered simultaneously, with age, sex, disease duration, and disease activity treated as confounders. Regression assumptions were checked before modelling, including multicollinearity (using variance inflation factor (VIF) values < 5), normality of residuals (Q-Q plots and Shapiro-Wilk test), homoscedasticity (residual-versus-fitted plots), and linearity (partial regression plots), with no major violations observed. A p-value <0.05 was considered statistically significant.

### Research ethics

The Institutional Review Board of Jinnah Hospital Lahore, Pakistan (Approval No. JHL/IRB/2025/317-A) approved this study before the data collection. All patients provided written informed consent, and the anonymity, confidentiality, and voluntary nature of their participation were guaranteed.

## Results

### Sociodemographic and clinical characteristics of participants

The demographic and clinical characteristics of the 504 patients with IBD are shown in Table 1. The majority of participants were young adults aged 18-24 years (45.6%) and female (54.4%). UC was more prevalent (59.3%) than CD (40.7%). The majority had a diagnosis duration of 4-6 years (40.7%) and reported low disease activity (37.1%). Just under half (45.4%) of the population had been admitted at least once in the last year, and corticosteroids were used most frequently (38.9%). Nearly half of the participants were married (44.2%), unemployed or retired (32.5%), and ex-smokers (44.4%).

**Table 1.** Participant Demographics (N = 504).

Variable	f (N)	%
Age group	-	-
18-24 years	230	45.6
25-34 years	160	31.7
35-44 years	70	13.9
45-54 years	25	5.0
55-64 years	12	2.4
65 years or above	7	1.4
Gender	-	-
Male	230	45.6
Female	274	54.4
Marital status	-	-
Single	105	20.8
Married	223	44.2
Divorced/separated	146	29.0
Widowed	30	6.0
Educational level	-	-
No formal education	125	24.8
Secondary school	158	31.3
College/ university	152	30.2
Post-graduate	69	13.7
Employment status	-	-
Student	146	29.0
Employed	92	18.3
Homemaker	77	15.3
Unemployed	164	32.5
Retired	25	5.0
Disease type	-	-
Crohn’s disease	205	40.7
Ulcerative colitis	299	59.3
Duration since diagnosis	-	-
<1 year	40	7.9
1-3 years	123	24.4
4-6 years	205	40.7
7-10 years	99	19.6
>10 years	37	7.3
Current disease activity (self-reported)	-	-
Remission (no symptoms)	133	26.4
Mild symptoms	187	37.1
Moderate symptoms	128	25.4
Severe symptoms	56	11.1
Hospitalization for IBD in the past year	-	-
None	151	30.0
Once	229	45.4
Twice or more	124	24.6
Current treatment	-	-
5-ASA (Mesalazine / Sulfasalazine)	84	16.7
Corticosteroids	196	38.9
Immuno-modulators	154	30.6
Biologics	57	11.3
None	13	2.6
Smoking status	-	-
Never smoked	171	33.9
Former smoker	224	44.4
Current smoker	109	21.6

f = frequency; % = percentage; IBD = inflammatory bowel disease; 5-ASA = 5-aminosalicylic acid; Values are presented as N (%), N = 504; No statistical comparisons were performed for demographic variables in this table.

### Normality test results for psychological and clinical variables

**Figure 1.**
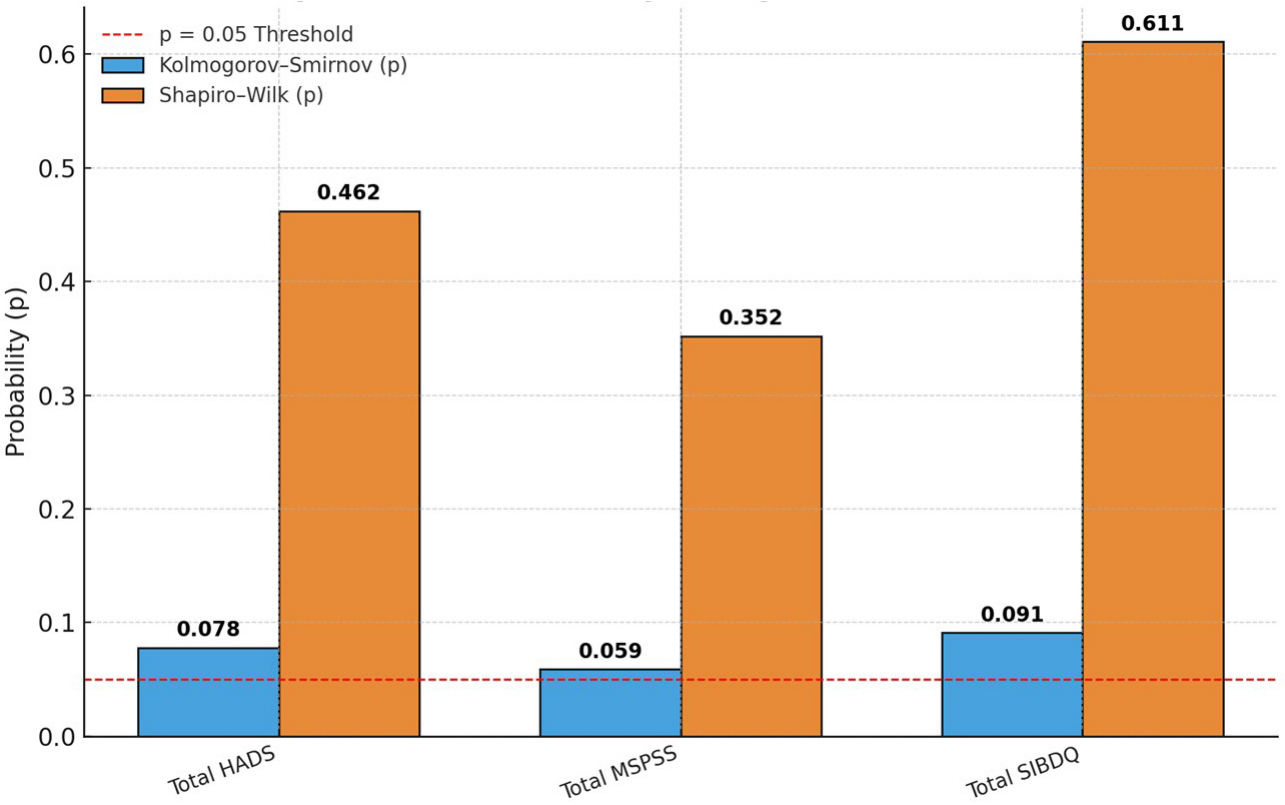
Normality test results for psychological and clinical variables (N = 504).

The normality test results (Kolmogorov-Smirnov and Shapiro-Wilk) for three psychological and clinical variables―HADS, MSPSS, and SIBDQ―are presented in Figure 1 (N = 504). The Shapiro-Wilk p-values for these three variables are all >0.05 (0.462 for HADS, 0.352 for MSPSS, and 0.611 for SIBDQ), indicating no significant departure from normality. The Kolmogorov-Smirnov p-values are somewhat smaller (all close to or just greater than 0.05) but indicative of reasonable normal approximation. Overall, this figure indicates that the data for all variables are approximately normally distributed, allowing the use of parametric tests.

### Correlation matrix of main study variables

Pearson’s correlations among psychological and clinical variables in 504 participants are demonstrated in Table 2. HADS scores showed an inverse correlation with MSPSS scores (r = −0.532, p < 0.001) and SIBDQ scores (r = −0.476, p < 0.001), suggesting that greater level of anxiety and depression is related to less perceived social support and poorer HRQL. In contrast, a strong positive association was observed between MSPSS and SIBDQ scores (r = 0.624, p < 0.001), suggesting that individuals with greater social support tend to report higher HRQL.

**Table 2.** Pearson Correlations and Corresponding t-Values among Psychological and Clinical Variables (N = 504).

Variables	r	df	t	p
Total HADS ↔ total MSPSS	−0.532	502	−14.07	<0.001^*^
Total HADS ↔ total SIBDQ	−0.476	502	−12.12	<0.001^*^
Total MSPSS ↔ total SIBDQ	0.624	502	17.89	<0.001^*^

Values represent Pearson correlation coefficients (r) between continuous variables; N = 504. HADS: Hospital Anxiety and Depression Scale; MSPSS: Multidimensional Scale of Perceived Social Support; SIBDQ: Short Inflammatory Bowel Disease Questionnaire. * = p < 0.001 was considered statistically significant.

### Gender-based differences in psychological distress, social support, and QOL

As shown in Table 3, psychological distress, perceived social support, and HRQL were compared across participants’ gender. Significant differences were noted in HADS scores between females (36.5 ± 4.9) and males (33.8 ± 4.2), indicating higher levels of psychological distress among females (t = −6.09, p < 0.001, d = 0.54). On the other hand, males tended to have a higher perception of social support (41.2 ± 6.4) than females did (39.3 ± 6.1) (t = 3.39, p < 0.001, d = 0.30). Likewise, men showed better HRQL, as indicated by higher SIBDQ scores (37.5 ± 5.1) than that shown by women (34.2 ± 5.4) (t = 6.33, p < 0.001, d = 0.56). Demographically, females had higher psychological distress and worse HRQL than males, but the pattern was reversed for social support.

**Table 3.** Group Differences by Gender in Psychological Distress, Social Support, and HRQL (N = 504).

Variable	Male (N = 230; 45.6%) M ± SD	Female (N = 274; 54.4%) M ± SD	t	p	95% CI (LL-UL)	Cohen’s *d*
Total Hospital Anxiety and Depression Scale (HADS)	33.8 ± 4.2	36.5 ± 4.9	−6.09	<0.001^***^	(−3.57 to −1.83)	0.54
Total Multidimensional Scale of Perceived Social Support	41.2 ± 6.4	39.3 ± 6.1	3.39	0.001^**^	(0.80 to 3.00)	0.30
Total Short Inflammatory Bowel Disease (IBD) Questionnaire	37.5 ± 5.1	34.2 ± 5.4	6.33	<0.001^***^	(2.28 to 4.32)	0.56

Values are presented as M ± SD; independent samples t-tests were conducted to compare participants of both genders; group sizes are shown as N (%); reported statistics include p-values, t-values, 95% confidence intervals (CIs), and effect sizes (Cohen’s d); ** = p < 0.01, *** = p < 0.001, N = 504. HRQL: health-related quality of life; LL: lower limit; M: mean; SD: standard deviation; UL: upper limit.

### Group differences between patients with CD and UC

Table 4 reports comparisons of psychological distress, perceived social support, and HRQL between patients with CD and UC. Patients with CD had significantly higher HADS scores (36.42 ± 3.65) than those with UC (33.89 ± 3.91), indicating greater psychological distress (t = 5.91, p < 0.001, d = 0.66). Conversely, patients with UC reported significantly more perceived social support (42.35 ± 6.12) than those with CD (39.12 ± 5.48) (t = −5.78, p < 0.001, d = 0.55). Similarly, patients with UC presented higher HRQL scores (36.04 ± 5.42) than those with CD (33.78 ± 5.16) (t = −4.75, p < 0.001, d = 0.43). Patients with CD had higher psychological distress and lower social support and HRQL compared to those with UC.

**Table 4.** Group Differences in Psychological Distress, Social Support, and HRQL between Patients with CD and UC (N = 504).

Variable	Crohn’s disease (N = 205; 40.7%) M ± SD	Ulcerative colitis (N = 299; 59.3%) M ± SD	t	p	95% CI (LL-UL)	Cohen’s d
Total Hospital Anxiety and Depression Scale (HADS)	36.42 ± 3.65	33.89 ± 3.91	5.91	<0.001^***^	(1.69 to 3.37)	0.66
Total Multidimensional Scale of Perceived Social Support	39.12 ± 5.48	42.35 ± 6.12	−5.78	<0.001^***^	(−4.33 to −2.13)	0.55
Total Short Inflammatory Bowel Disease (IBD) Questionnaire	33.78 ± 5.16	36.04 ± 5.42	−4.75	<0.001^***^	(−3.20 to −1.32)	0.43

Values are presented as M ± SD; independent samples t-tests were conducted to compare participants of both diseases; group sizes are shown as N (%); reported statistics include p-values, t-values, 95% confidence intervals (CIs), and effect sizes (Cohen’s d); *** = p < 0.001, N = 504. CD: Crohn’s disease; HRQL: health-related quality of life; LL: lower limit; M: mean; SD: standard deviation; UC: ulcerative colitis; UL: upper limit.

### Associated factors of HRQL among patients with IBD

Table 5 presents the associations of psychological distress, social support, and clinical variables with HRQL measured by SIBDQ in 504 patients with IBD. Higher psychological distress (HADS; B = −0.412, 95% confidence intervals [CIs]: −0.527 to −0.296, p < 0.001) and greater self-reported disease activity (B = −1.085, 95% CI: −1.533 to −0.637, p < 0.001) were significantly associated with lower HRQL. Conversely, higher perceived social support (MSPSS; B = 0.268, 95% CI: 0.198-0.338, p < 0.001) and older age (B = 0.215, 95% CI: 0.074-0.356, p = 0.003) were associated with higher HRQL. Female gender (B = −3.624, 95% CI: −5.808 to −1.440, p = 0.001), longer duration since diagnosis (B = −0.286, 95% CI: −0.506 to −0.066, p = 0.011), and a diagnosis of CD (B = −1.472, 95% CI: −2.438 to −0.506, UC coded as 1, CD coded as 2, p = 0.003) were also linked to lower HRQL scores. Among these, the female gender showed the most significant negative association with HRQL. Overall, psychological, social, and clinical factors were associated with variations in HRQL among patients with IBD.

**Table 5.** Multiple Linear Regression of HRQL Measured by the SIBDQ on Psychological Distress, Social Support, and Clinical Variables.

Predictor	B	SE	β	t	p	95% CI (LL-UL)
Constant (SIBDQ)	18.742	3.920	―	4.78	< 0.001***	(10.997 to 26.487)
Total Hospital Anxiety and Depression Scale (HADS)	−0.412	0.058	−0.289	−7.10	< 0.001***	(−0.527 to −0.296)
Total Multidimensional Scale of Perceived Social Support (MSPSS)	0.268	0.036	0.302	7.44	< 0.001***	(0.198 to 0.338)
Age	0.215	0.072	0.111	2.99	0.003**	(0.074 to 0.356)
Gender (1 = male, 2 = female)	−3.624	1.112	−0.143	−3.26	0.001**	(−5.808 to −1.440)
Duration since diagnosis	−0.286	0.112	−0.094	−2.55	0.011*	(−0.506 to −0.066)
Current disease activity (self-reported)	−1.085	0.228	−0.210	−4.76	< 0.001***	(−1.533 to −0.637)
Disease type (UC = 1, CD = 2)	−1.472	0.491	−0.098	−3.00	0.003**	(−2.438 to −0.506)

Multiple linear regression was conducted to identify associated factors scores; age and duration since diagnosis are measured in years; gender coded as 1 = male and 2 = female; disease type coded as 1 = ulcerative colitis and 2 = Crohn’s disease; Values include unstandardized coefficients (B), 95% confidence intervals (CIs), standard error (SE), standardized beta coefficients (β), and p-values; * = p < 0.05, **=p < 0.01, ***=p < 0.001 was considered statistically significant, N = 504. CD: Crohn’s disease; HRQL: health-related quality of life; LL: lower limit; SIBDQ: Short Inflammatory Bowel Disease Questionnaire; UC: ulcerative colitis; UL: upper limit.

### Unstandardized regression coefficients for associated factors of HRQL

**Figure 2.**
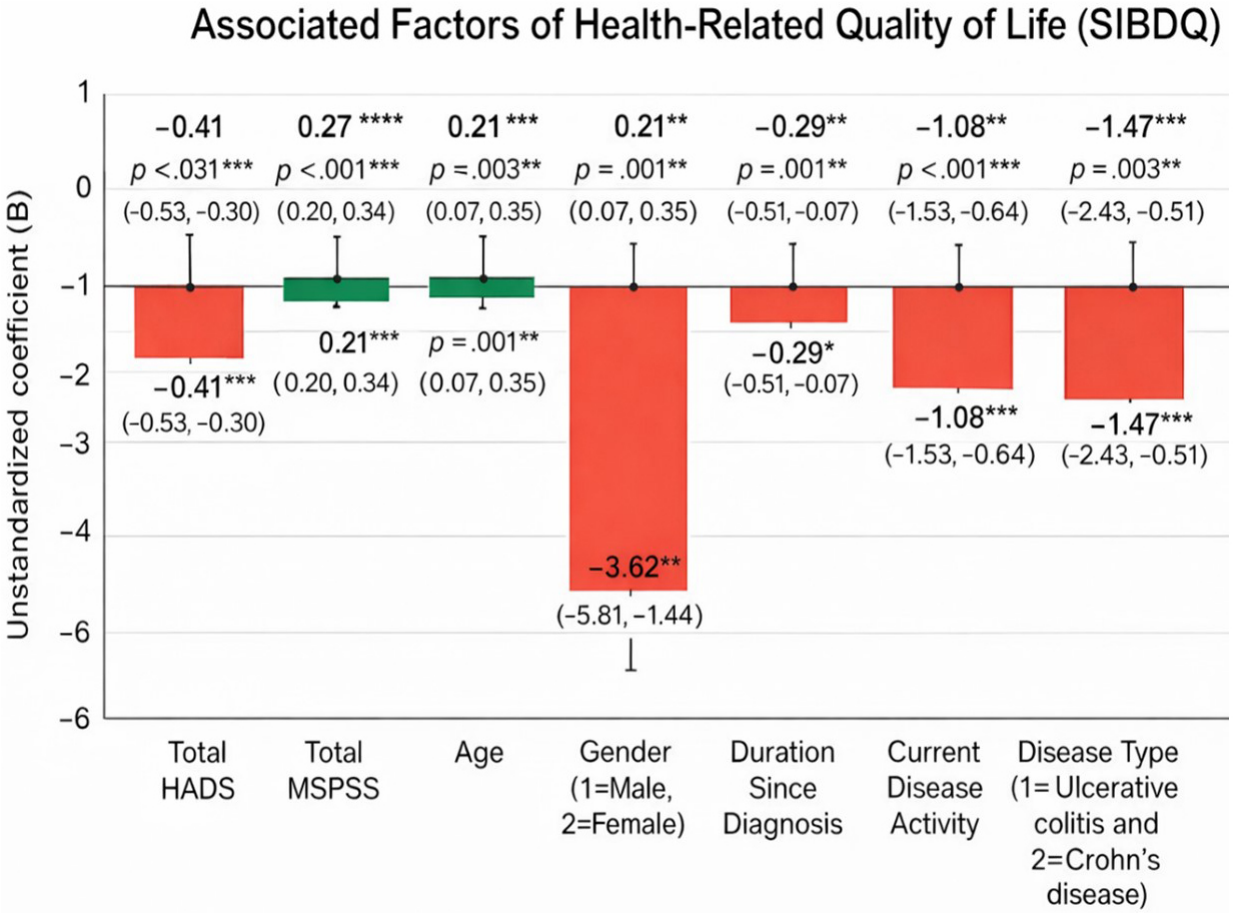
Associated factors of health-related quality of life (HRQL) measured by the Short Inflammatory Bowel Disease Questionnaire (SIBDQ) among patients with inflammatory bowel disease (IBD).

Figure 2 illustrates the direction of associations between key psychological, social, and clinical factors and HRQL among patients with IBD. Psychological distress, female gender, longer disease duration, active disease status, and CD diagnosis were negatively associated with HRQL, indicating poorer perceived well-being in these groups. In contrast, greater perceived social support and older age were positively associated with HRQL, suggesting a protective role of social resources and adaptive coping with age. Overall, the figure highlights the multifactorial nature of HRQL in IBD, emphasizing the combined influence of psychological, social, and disease-related factors.

## Discussion

In this study, we investigated the relationships among total HADS score, MSPSS scores, and HRQL scores for individuals with IBD. In this study, there was a negative correlation between social support and psychological distress, with participants who had higher total HADS scores reported lower MSPSS scores. Similar results have been reported in previous research, which emphasized the protective role of social support in alleviating psychological distress, even when controlling for burnout ^(14)^. Elevated psychological distress was significantly associated with worse HRQL in this study. This result is in line with earlier evidence showing that higher baseline emotional symptom burden was associated with lower HRQL among patients with a chronic illness and that positive coping strategies were associated with better well-being ^(15)^. Conversely, better perceived social support was associated with higher HRQL, corroborating earlier studies that showed social support acts as a protective factor by alleviating psychological distress and promoting well-being ^(16)^.

There were also differences between genders, with women reporting greater psychological distress and lower HRQL compared with those reported in men. The findings are consistent with those reported in the literature that have indicated higher vulnerability to emotions and functional impairment in female patients ^(17), (18)^. Males had significantly better perceived social support than females in this study. Nevertheless, previous research has yielded mixed results, suggesting that women tend to have higher-quality social support and larger social networks. This disparity might indicate that men and women experienced and employed social relationships differently, or that they reported social relationships differently across populations and cultural settings ^(19)^. These gender differences may reflect psychosocial factors, such as differential coping strategies, societal expectations, or caregiving responsibilities, which can influence perceived support and emotional burden. Similarly, the higher psychological distress observed in patients with CD may be related to the unpredictable disease course, presence of perianal disease, or stricter dietary restrictions, which could exacerbate emotional symptom burden and reduce HRQL.

In our sample, patients with CD exhibited higher total HADS scores than those with UC, suggesting greater emotional symptom burden in CD. These findings are parallel with prior studies where CD was associated with increased psychological distress, potentially because of its unpredictable course and a greater severity of symptom burden ^(20)^. Patients with UC in our sample reported higher MSPSS scores than those with CD, indicating greater perceived social support. This contrasts with earlier studies that showed no significant difference in social support between the two patient groups, suggesting that social and emotional resources differ across populations or cultures ^(7)^. Similarly, HRQL was higher in patients with UC than in those with CD in our sample, suggesting a greater negative impact of CD on disease-specific HRQL. Comparable results were reported in a recent study, which found that patients with CD had poorer overall HRQL than those with UC ^(21)^.

Psychological distress, social support, age, and gender were the most critical factors affecting HRQL in patients with IBD in the regression analysis. Higher total HADS scores were associated with lower HRQL, indicating that greater emotional symptom burden was linked to worse outcomes; lower MSPSS scores were also associated with lower HRQL. Older age was associated with higher HRQL, whereas females had lower HRQL than males, as previously found ^(15), (18), (22)^. In our study, a longer time since IBD diagnosis was weakly but significantly associated with lower HRQL, which is in agreement with other research showing that patients over time often have more psychological burden and lower well-being ^(23)^. Higher self-reported disease activity was associated with lower HRQL, consistent with prior literature showing worse HRQL in active versus quiescent patients with IBD ^(24)^. Patients with CD had slightly lower HRQL than those with UC, consistent with the greater impact on well-being and daily function observed in previous studies ^(21)^.

### Study constraints and recommendations

Several limitations should be considered. First, the cross-sectional design does not allow for causal inference so that no definitive directionality can be established among psychological distress, perceived social support, and HRQL. Second, self-report questionnaires (HADS, MSPSS, and SIBDQ) were used to gather data, which may introduce response or recall bias and shared-method variance. Third, the convenience sampling of outpatient clinics in two urban areas limits generalizability, restricting the applicability of findings to other populations, rural environments, or patients with more severe disease. Fourth, disease activity was assessed solely via self-report without using validated indices or clinician-rated measures, such as the Harvey-Bradshaw Index (HBI), the Simple Clinical Colitis Activity Index (SCCAI), or endoscopic findings, which represents a substantial methodological limitation. Fifth, the omission of hospitalized or acutely ill patients may have underestimated the actual psychological burden of IBD in the population. Finally, the study did not examine multicollinearity or regression model assumptions (linearity, homoscedasticity, and residual normality), which should be explicitly checked in future research.

Future research should utilize longitudinal designs to clarify the temporal relationship among psychological distress, social support, and HRQL. The inclusion of clinical biomarkers and physician-rated disease activity can enhance validity and provide a more comprehensive view of biopsychosocial processes in IBD. Interventional studies assessing the effects of psychological therapies, peer support groups, or family-based interventions on HRQL are warranted. Additionally, examining gender- and age-specific coping mechanisms can help design targeted psychosocial interventions for at-risk subgroups, such as young adults and women with CD.

### Conclusions

This study examined the associations among HRQL, perceived social support (MSPSS), psychological distress (total HADS), and clinical factors in patients with IBD. Higher psychological distress and greater self-reported disease activity were associated with lower HRQL, whereas higher perceived social support and older age were associated with better HRQL. Female gender, longer disease duration, and diagnosis of CD were also linked to lower HRQL. These findings indicate that psychological, social, and clinical factors are associated with variations in HRQL, though the cross-sectional design does not allow causal conclusions. The results underscore the importance of considering psychological screening and social support interventions alongside pharmacological management to improve HRQL in patients with IBD.

## Article Information

### Author Contributions

Purvi Sukhwal conceptualized and supervised the study. Ghazanfar Aftab contributed to study design and data collection. Minal Tariq conducted the statistical analysis and interpreted the results. Daniah Hisham Al-Sammarraie managed patient recruitment and clinical validation. Alhasan Hisham Al-Sammarraie and Zainab Hisham Al-Sammarraie contributed to the literature review and drafting. Afnan Ahmed and Muhammad Saad Ullah revised and formatted the manuscript. Abdulrahman AlQaderi provided methodological guidance and reviewed clinical implications. Maheen Haque coordinated manuscript preparation. Hayian Omran supervised the project, oversaw manuscript revisions, and is the corresponding author. All authors read and approved the final version of the manuscript.

### Conflicts of Interest

None

### IRB Approval Code and Institution

This study was approved by the Institutional Review Board of Jinnah Hospital Lahore, Pakistan (Approval No. JHL/IRB/2025/317-A).

## References

[1] Zhang YZ, Li YY. Inflammatory bowel disease: pathogenesis. World J Gastroenterol. 2014;20(1):91-9. 24415861 10.3748/wjg.v20.i1.91 PMC3886036

[2] Ng SC, Shi HY, Hamidi N, et al. Worldwide incidence and prevalence of inflammatory bowel disease in the 21st century: a systematic review of population-based studies. Lancet. 2017;390(10114):2769-78. 29050646 10.1016/S0140-6736(17)32448-0

[3] Yildirim Y, Artan Y, Unal NG. Relationship between social isolation, loneliness and psychological well-being in inflammatory bowel disease patients: the mediating role of disease activity social isolation in inflammatory bowel disease. J Clin Nurs. 2024;34(8):3276-87. 39468919 10.1111/jocn.17509

[4] Knowles SR, Wilson J, Wilkinson A, et al. Psychological well-being and quality of life in Crohn’s disease patients with an ostomy: a preliminary investigation. J Wound Ostomy Continence Nurs. 2013;40(6):623-9. 24202226 10.1097/WON.0b013e3182a9a75b

[5] Zhang M, Hong L, Zhang T, et al. Illness perceptions and stress: mediators between disease severity and psychological well-being and quality of life among patients with Crohn’s disease. Patient Prefer Adherence. 2016;10:2387-96. 27920505 10.2147/PPA.S118413 PMC5125764

[6] Sewitch MJ, Abrahamowicz M, Bitton A, et al. Psychological distress, social support, and disease activity in patients with inflammatory bowel disease. Am J Gastroenterol. 2001;96(5):1470-9. 11374685 10.1111/j.1572-0241.2001.03800.x

[7] Slonim-Nevo V, Sarid O, Friger M, et al. Effect of social support on psychological distress and disease activity in inflammatory bowel disease patients. Inflamm Bowel Dis. 2018;24(7):1389-400. 29893949 10.1093/ibd/izy041

[8] Palant A, Himmel W. Are there also negative effects of social support? A qualitative study of patients with inflammatory bowel disease. BMJ Open. 2019;9(1):e022642. 10.1136/bmjopen-2018-022642 PMC6347904 30670504

[9] Knowles SR, Keefer L, Wilding H, et al. Quality of life in inflammatory bowel disease: a systematic review and meta-analyses-part II. Inflamm Bowel Dis. 2018;24(5):966-76. 29688466 10.1093/ibd/izy015

[10] Fu H, Kaminga AC, Peng Y, et al. Associations between disease activity, social support and health-related quality of life in patients with inflammatory bowel diseases: the mediating role of psychological symptoms. BMC Gastroenterol. 2020;20(1):11. 31937264 10.1186/s12876-020-1166-y PMC6961247

[11] Zigmond AS, Snaith RP. The hospital anxiety and depression scale. Acta Psychiatr Scand. 1983;67(6):361-70. 6880820 10.1111/j.1600-0447.1983.tb09716.x

[12] Zimet GD, Dahlem NW, Zimet SG, et al. The Multidimensional Scale of Perceived Social Support. J Pers Assess. 1988;52(1):30-41.

[13] Jowett SL, Seal CJ, Barton JR, et al. The short inflammatory bowel disease questionnaire is reliable and responsive to clinically important change in ulcerative colitis. Am J Gastroenterol. 2001;96(10):2921-8. 11693327 10.1111/j.1572-0241.2001.04682.x

[14] Sánchez-Moreno E, de La Fuente Roldán IN, Gallardo-Peralta LP, et al. Burnout, informal social support and psychological distress among social workers. Br J Soc Work. 2015;45(8):2368-86.

[15] Ramirez SP, Macêdo DS, Sales PMG, et al. The relationship between religious coping, psychological distress and quality of life in hemodialysis patients. J Psychosom Res. 2012;72(2):129-35. 22281454 10.1016/j.jpsychores.2011.11.012

[16] Atkins J, Naismith SL, Luscombe GM, et al. Psychological distress and quality of life in older persons: relative contributions of fixed and modifiable risk factors. BMC Psychiatry. 2013;13(1):249. 24103220 10.1186/1471-244X-13-249 PMC3852717

[17] Viertiö S, Kiviruusu O, Piirtola M, et al. Factors contributing to psychological distress in the working population, with a special reference to gender difference. BMC Public Health. 2021;21(1):611. 33781240 10.1186/s12889-021-10560-y PMC8006634

[18] Pandelani FF, Nyalunga SLN, Mogotsi MM, et al. Chronic pain: its impact on the quality of life and gender. Front Pain Res (Lausanne). 2023;4:1253460. 37781217 10.3389/fpain.2023.1253460 PMC10534032

[19] Kneavel M. Relationship between gender, stress, and quality of social support. Psychol Rep. 2020;124(4):1481-501. 32635816 10.1177/0033294120939844

[20] Song JH, Kim JW, Oh CH, et al. Depression, anxiety, related risk factors and cognitive distortion in Korean patients with inflammatory bowel disease. Psychiatry Investig. 2020;17(11):1126-36. 10.30773/pi.2020.0299 PMC7711122 33115188

[21] Keller R, Mazurak N, Fantasia L, et al. Quality of life in inflammatory bowel diseases: it is not all about the bowel. Intest Res. 2021;19(1):45-52. 32093437 10.5217/ir.2019.00135 PMC7873402

[22] Pizzi LT, Weston CM, Goldfarb NI, et al. Impact of chronic conditions on quality of life in patients with inflammatory bowel disease. Inflamm Bowel Dis. 2006;12(1):47-52. 16374258 10.1097/01.mib.0000191670.04605.e7

[23] Bernabeu P, Belén-Galipienso O, van-der Hofstadt C, et al. Psychological burden and quality of life in newly diagnosed inflammatory bowel disease patients. Front Psychol. 2024;15:1334308. 38348263 10.3389/fpsyg.2024.1334308 PMC10859525

[24] Armuzzi A, Tarallo M, Lucas J, et al. The association between disease activity and patient-reported outcomes in patients with moderate-to-severe ulcerative colitis in the United States and Europe. BMC Gastroenterol. 2020;20(1):18. 31964359 10.1186/s12876-020-1164-0 PMC6975026

